# Hyperandrogenism? Increased 17, 20-Lyase Activity? A Metanalysis and Systematic Review of Altered Androgens in Boys and Girls with Autism

**DOI:** 10.3390/ijms222212324

**Published:** 2021-11-15

**Authors:** Benedikt A. Gasser, Samuel F. Buerki, Johann Kurz, Markus G. Mohaupt

**Affiliations:** 1 Departement für Sport, Bewegung und Gesundheit, University of Basel, 4001 Basel, Switzerland; 2 Lindenhofspital Teaching Hospital Internal Medicine, 3001 Bern, Switzerland; samuel.buerki313@gmail.com (S.F.B.); markus.mohaupt@lindenhofgruppe.ch (M.G.M.); 3 Interscience Research Association, 8430 Leibnitz, Austria; john.kurz@a1.net

**Keywords:** extreme male brain theory of autism, androgens, steroid hormones

## Abstract

*Introduction:* There is increasing evidence that steroid hormone levels and, especially, androgen levels are elevated in autism. An overactivity of 17, 20-lyase with a higher production of the testosterone precursors dehydroepiandrosterone (DHEA) and androstenedione/androstenediol seems especially present in autism. *Methods:* An encompassing literature analysis was performed, searching for altered androgens in children with autism and using preferred reporting items for systematic reviews and meta-analysis (PRISMA) guidelines. Included were all studies published before 31 March 2021 found using the following electronic databases: PubMed, Google Scholar, Cochrane Library, Scopus, and TRIP. Eight studies with boys and three studies with girls where steroid hormone measurements were performed from either plasma, urine, or saliva were found and analyzed. Analyses were performed for DHEA(-S/-C), androstenedione/androstenediol, and testosterone. Effect sizes were calculated for each parameter between mean concentrations for children with autism versus healthy controls. *Results:* Higher levels of androgens in autism were detected, with the majority of calculated effect sizes being larger than one. *Conclusions:* We found higher levels of the main testosterone precursors DHEA, androstenedione, and androstenediol, likely causing an additionally higher level of testosterone, and an increased 17, 20-lyase activity is therefore implied. Medications already used in PCOS such as metformin might be considered to treat hyperandrogenism in autism following further research.

## 1. Introduction

Endocrine abnormalities are often suggested to be possible etiopathogenetic causes of autism. In the original work of Hans Asperger, the four cases of Fritz, Hellmuth, Harro, and Ernst were analyzed and described with regard to endocrine abnormalities. The involvement of the HPA axis and cholesterol as precursors of steroid hormones as well as sex hormones, androgens in particular, due to their higher prevalence in boys than in girls, were suggested to be altered in children with autism [1,2]. Case reports concerning hypermasculinization in the 1980s and reports about precocious puberty followed [3]. Further associations between elevated androgen levels and fragile X syndrome as well as autism were implied [3] and, as a consequence, autism has been linked with hormonal disbalance for a long time [1,4]. One theory directly addressing steroid hormone change and the higher prevalence in boys than in girls is known as extreme male brain theory [5]. Further analyses have focused on altered steroid hormones while also addressing enzymatic activities and different forms of hormone regulation over the lifespan [6,7,8]. Moreover, in addition to studies directly analyzing androgens from urine, saliva, serum/plasma, and amniotic fluid, clear associations between changes in androgen levels and autism have been observed. Xu et al. (2013) measured higher testosterone levels in mothers of children with autism [9]. Ingudomnukul detected elevated rates of testosterone-related disorders in women with autism [10]. Takasaki et al. correlated salivary testosterone levels with severity of autism, indicating a positive association [11]. Palomba et al. showed that daughters of mothers affected by hyperandrogenic PCOS seem to be at a high risk of autism, possibly due to an unbalanced prenatal exposure to high levels of testosterone [12]. Saenz et al. showed that postnatal testosterone levels were related to autistic behavior in the second year of life [13]. Other studies have elucidated the involvement of the HPA axis and steroid hormones in autism [14]. As the CRH–ACTH system and cortisol are the main mediators of stress, a whole line of evidence acknowledges their variation in children with autism (review by Taylor and Corbett, 2014) [14]. The HPA axis, and its dysregulation in autism in particular, has thus been broadly discussed [9,15,16,17,18,19,20,21,22]. Differences have been found on the level of the hypothalamus [15,16], the pituitary gland [21,23,24,25], and the adrenal gland [5,24].

A further line of evidence was derived from the cholesterol hypothesis of autism and suggests an increase in cholesterol, the main precursor of steroid hormones [2]. As a result, once again, special attention was given to sex hormones and androgens [5]. Newer studies have directly searched for an explanation for the higher levels of sex hormones, with one line of evidence suggesting retinoic orphan receptor A (RORa) as a key mediator [25,26,27,28]. RORa regulates more than 600 genes in the human genome, many of them affecting neuronal plasticity and memory formation; it is differently activated by androgens and estrogens, thereby directly explaining the higher prevalence in boys than in girls [25,26,27,28]. Furthermore, the role of RORa regulating aromatase, the enzyme that transforms testosterone to estrogens, has been implied [25,26,27,28]. Direct measurements have shown that the aromatase protein is significantly reduced in the frontal cortex of subjects and is strongly correlated with RORa protein levels in the brain [25,26,27,28]. However, the detailed mechanism of testosterone predisposing autism remains unclear [6,7]. Genetic research alone has not provided a profound understanding of the underlying causes, and a detailed molecular understanding of the involved pathways is missing [29]. To date, it has been confirmed that testosterone easily passes the blood–brain barrier, and its receptor, the androgen receptor, is expressed in various brain cells and regions [30,31]. In rodents, androgen receptors are expressed in brain areas that are important for the regulation of emotions, cognition, and behavior. Androgens also play a major role in establishing anatomical and functional sexual dimorphism in the nervous system [31,32,33]. Furthermore, several studies have demonstrated that early exposure to testosterone or to its more active metabolite, dihydrotestosterone, provides neuroprotection [31,34,35] and allows for the modulation of synaptic density and neurite outgrowth in some brain regions [31,36,37].

**Figure 1:**
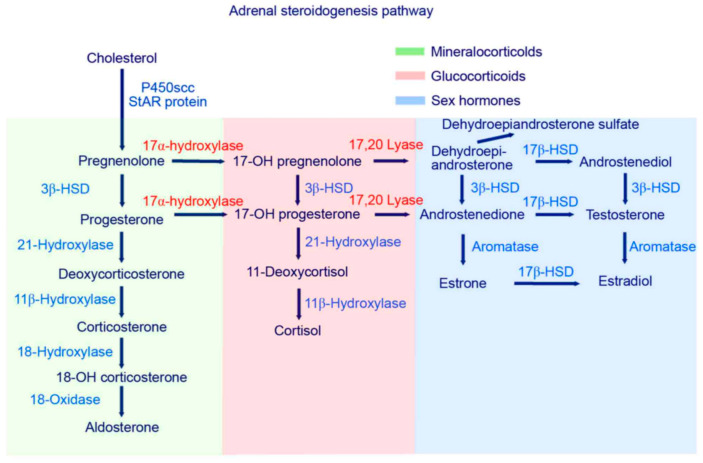
Steroid hormone activity—higher 17, 20-lyase activity should be indicated by higher levels of DHEA, DHEA-S and androstenedione. One step further, higher levels of androstenediol and testosterone in line with increased 17B-HSD and 3B-HSD are indicative of a general hyperandrogenism in autism [39].

In conclusion, there are various suggestions of the involvement of altered androgen levels in autism. Further cohort studies imply an overactivity of 17, 20-lyase with higher levels of the testosterone precursors DHEA, androstenedione, and androstenediol found in children with autism (Figure 1). The aim of the current study is a direct product of the above discussion: to systematically analyze and summarize the current evidence of higher androgen levels in children with autism. As a hypothesis with potential falsification, it is stated that DHEA, androstenedione, androstenediol, and testosterone concentrations are not higher in serum/plasma, urine, or saliva in children with autism as compared to those of healthy controls [38].

## 2. Materials and Methods

### 2.1. Search Strategy

**Figure 2:**
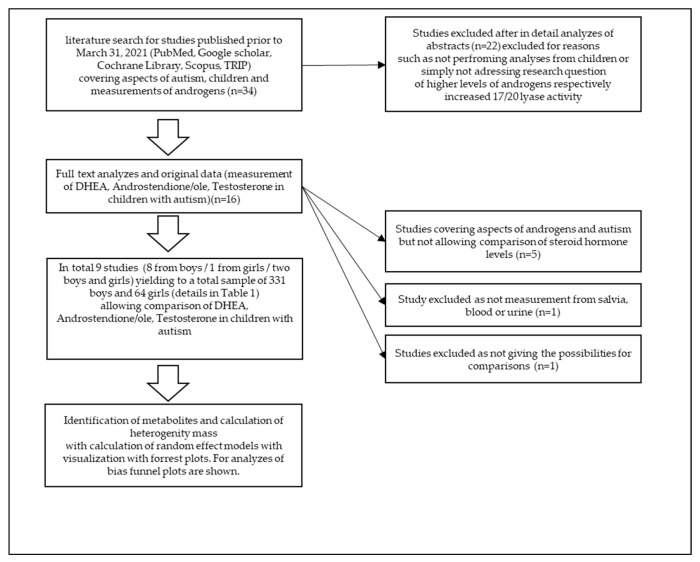
Flow diagram of meta-analysis procedure.

This meta-analysis (Figure 2) was performed using preferred reporting items for systematic reviews and meta-analysis (PRISMA) guidelines. A comprehensive literature search for studies published prior to 31 March 2021 was conducted using the following electronic databases: PubMed, Google Scholar, Cochrane Library, Scopus, and TRIP. The following search terms were employed: “androgens” OR “autism” OR “children” OR “Testosterone” AND/OR “DHEA” AND/OR “Androstenedione” AND/OR “Androstenediole” AND “children” AND “boys” AND “girls” OR “ASD” OR “autism”. The literature cited by included studies was also manually searched for additional eligible studies.

### 2.2. Study Eligibility Criteria

Studies involving children with autism diagnosed according to current guidelines (e.g., DSM-IV/V/ICD-10) undergoing analyses of steroid hormones from plasma/serum, urine, or saliva were eligible and included.

### 2.3. Data Collection and Analysis

All eligible studies were screened by two independent reviewers using the selection criteria listed above. Screening first entailed abstract review, followed by full-text review. Any discrepancies were settled through discussion. The following information was extracted from each included study: number of girls and boys with autism and potential healthy controls, levels of steroid hormones (total of all potential steroid hormones measured with potentially androgenic activity, androstenedione, androstenediol, DHEA, and testosterone), and probe material (urine, serum/plasma, and saliva).

### 2.4. Outcomes

The primary outcome evaluated in this study was increased measured androgen concentrations in children with autism as compared to those in healthy controls in either serum/plasma, urine, or saliva fluid.

### 2.5. Publication Bias

Potential publication bias was assessed using funnel plot analysis [40].

### 2.6. Statistical Analysis

Effect sizes were calculated as the difference in mean change between the ASD group and the comparison group divided by the pooled standard deviation (Hedge’s *g*) [41]. For the studies of Geier and Geier (2006 and 2007), only reference values and no control group were reported. As such, the difference between the reference values and the measured values in combination with the respective standard deviation was taken in order to calculate effect sizes. Effect sizes were interpreted as small for d = 0.2, moderate for d = 0.5, and large for d = 0.8 or more. Mean differences (MDs) with 95% confidence intervals (CIs) were calculated for the reported steroid hormone levels for the respective subsample in girls and boys with autism versus unaffected controls. Cochrane’s Q statistic provides a measure of the variance between the effect sizes (with *p* < 0.05 illustrating evidence of heterogeneity), while I^2^ provides a measure of the amount of variance between studies in terms of heterogeneity [41,42,43], whereby I^2^ > 50% indicates substantial heterogeneity [41]. Here, the heterogeneity variance was estimated using the restricted maximum likelihood method (REML) [42,43]. As study heterogeneity was observed in all the metabolites analyzed except for testosterone in girls with an I^2^ value larger than 0.5, random effects models were applied [41,42,43]. Statistical analyses were conducted using Review Manager software (Version 5.3, Copenhagen: The Nordic Cochrane Center, The Cochrane Collaboration 2014) and R (version 4.1.1) [43].

## 3. Results and Discussion

Table 1 provides an overview of the studies performing measurements of steroid hormones in blood, urine, or saliva. In total, eight studies on boys were included, with a total sample of 331 boys and 64 girls with autism (Table 1). Twenty years ago, Tordjman et al. (1995) performed an analysis of altered levels of testosterone and the sulfated androgen DEHA-S in plasma, their findings indicating that significantly higher levels of these hormones could not be found in children with autism as compared to healthy controls [3]. This was followed by two studies by Geier and Geier (2006, 2007) [44,45]. El-Baz measured serum androgen levels in a group of Egyptian male autistic children and adolescents and their relation to disease severity, where the results showed, in addition to higher androgen levels, an association between disease severity and androgen levels [46]. Croonenberghs et al. analyzed serum testosterone concentrations over time (one day—nine measurements) in male autistic children and detected, in contrast to most other studies, lower levels of testosterone in subjects with autism [47]. Majewska et al. distinguished between prepubertal and pubertal children [48] and compared the salivary levels of 22 steroids in prepubertal autistic male and female children from two age groups (3–4 and 7–9 years old) with those in healthy controls. Children with autism had significantly higher salivary concentrations of androgens (androstenediol, DHEA, androsterone, and their polar conjugates) than those of healthy controls. Ruta et al. (2011) measured the three androgens testosterone, androstenedione, DHEA sulfate and found high levels [23]. Gasser et al. measured around 40 metabolites from urine in boys and girls with autism using GC–MS [7,49]. Furthermore, Janšáková et al. (2020) [6] performed measurements from blood using GC–MS analysis of samples of boys with autism, and alterations in steroid hormones and notably higher androgens in boys with autism before puberty compared to healthy controls were observed.

**Figure 3:**
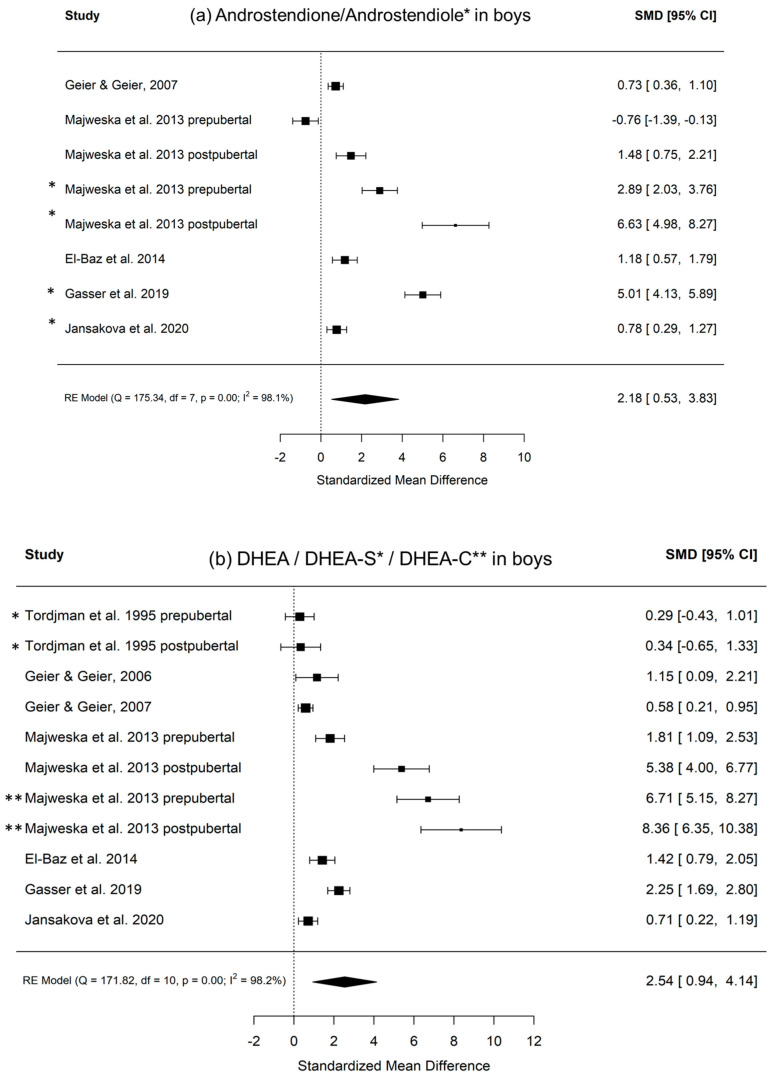

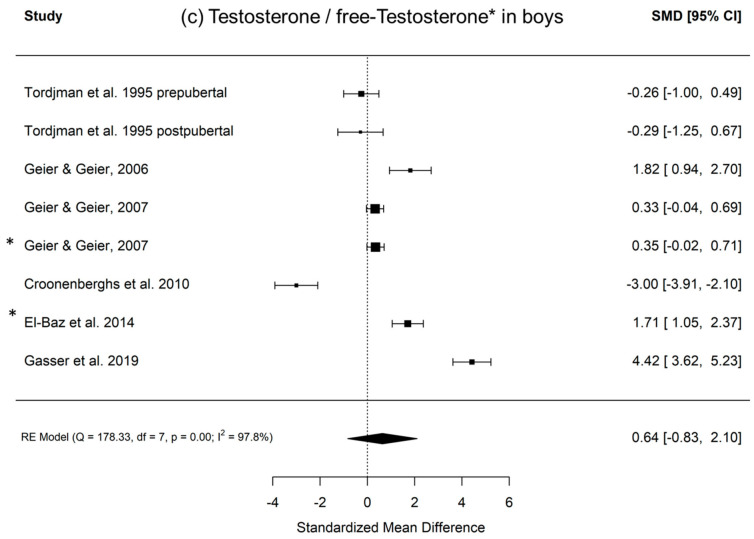
(**a**–**c**) Random effects models with significance level, Cochrane Q, I^2^, and effect sizes of analyzed metabolites in boys with autism versus healthy controls. (**a**) Androstenedione/androstenediol indicated with *; (**b**) DHEA/DHEA-S indicated with *, DHEA-C indicated with **; (**c**) testosterone/free testosterone indicated with *.

**Figure 4:**
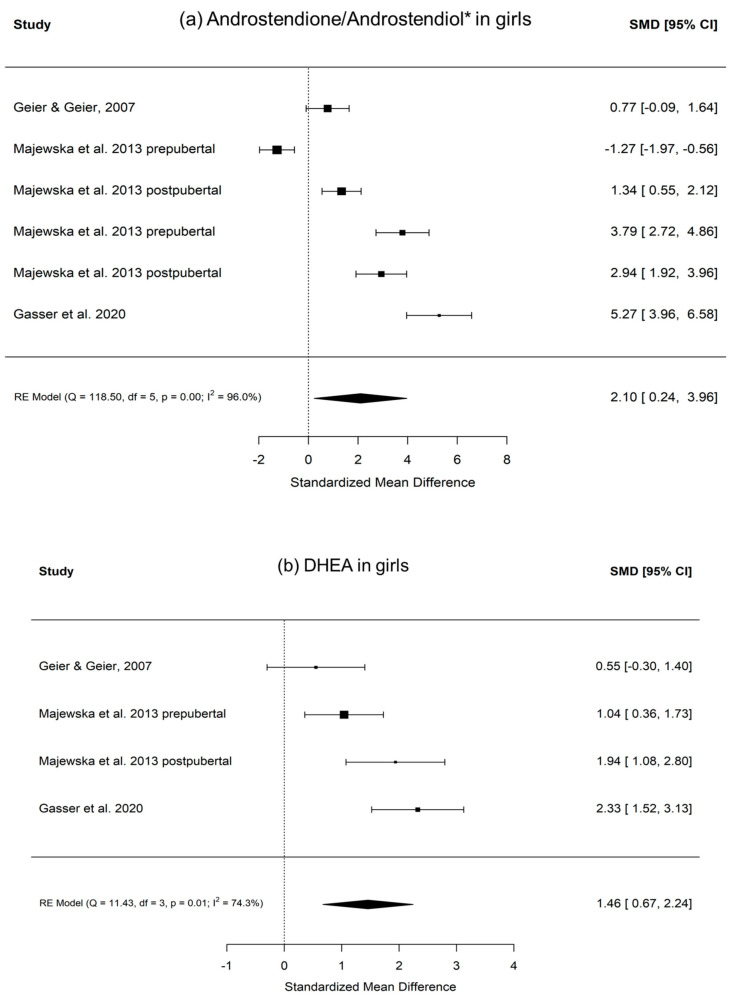

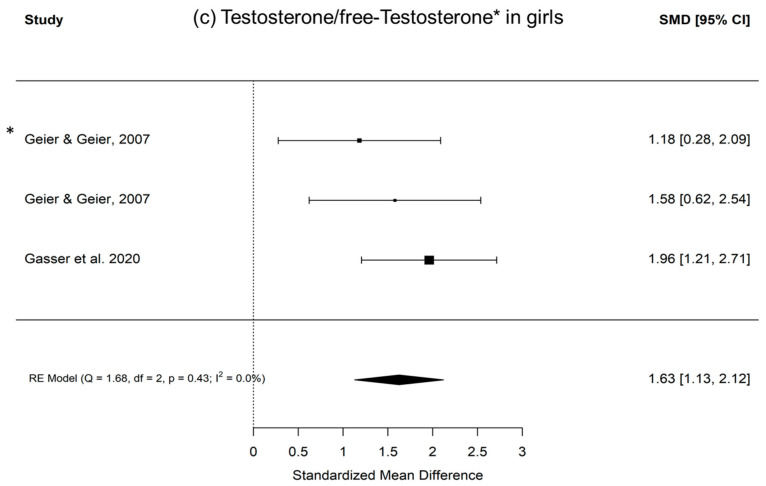
(**a**–**c**) Random effects models with significance level, Cochrane Q, I^2^, and effect sizes of analyzed metabolites in girls with autism versus healthy controls. (**a**) Androstenedione/androstenediol indicated with *; (**b**) DHEA; (**c**) testosterone/free testosterone indicated with *.

**Figure 5:**
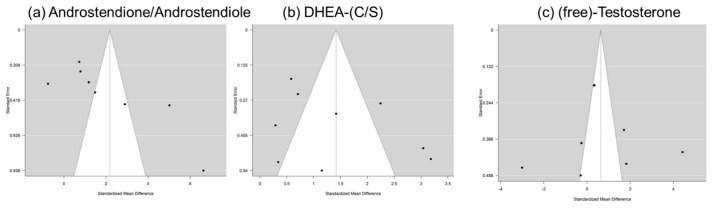
Funnel plots for boys: *x*-axis—standardized mean difference; *y*-axis—standard error for (**a**) androstenedione/androstenediol, (**b**) DHEA-(C/S), and (**c**) (free) testosterone.

**Figure 6:**
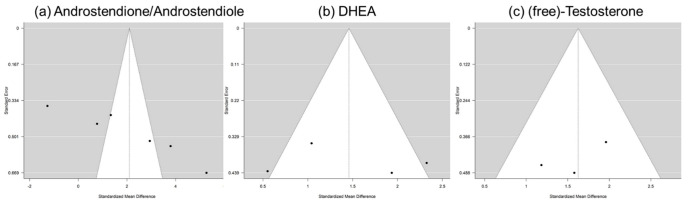
Funnel plots for girls: *x*-axis—standardized mean difference; *y*-axis—standard error for (**a**) androstenedione/androstenediol, (**b**) DHEA-(C/S), and (**c**) (free) testosterone.

Figure 3 shows the calculated random effects models for boys with autism versus controls for androstenedione/androstenediol, DHEA, and testosterone. Figure 4 shows the calculated random effects models for girls with autism versus healthy controls for androstenedione/androstenediol, DHEA, and testosterone. Furthermore, Figure 5 and Figure 6 show the funnel plots for boys and girls, respectively.

In summary, the aim of this review was to discuss the evidence of altered androgens, with a special focus on 17, 20-lyase activity in girls and boys with autism. The initially stated hypothesis that DHEA, androstenedione, androstenediol, and testosterone levels are not higher in subjects with autism than those in healthy seems rejectable. The general consensus is that androgen levels are higher in children with autism than in healthy controls. As only evidence from children is shown, the development over the lifespan remains indicative. Nevertheless, the study conducted by Ruta et al. (2011) [23], with a relatively large sample of 58 adult subjects (the average age was around 30 years) with autism versus 70 controls, showed significantly increased levels of androstenedione in the adult subjects with autism. However, the levels of DHEA sulfate, free testosterone, and total testosterone in those with autism were not observed to be significantly higher than those in the controls. From the eight analyzed studies focusing on boys in detail, only the oldest one conducted by Tordjman et al. in 1995 and the study by Croonenberghs et al. in 2010 report low androgens in children with autism [47], and the findings by Tordjman et al. in 1995 might be influenced by the relatively heterogeneous nature of the sample [3]. The findings report that mean levels of plasma testosterone and DHEA sulfate were similar in pre- and post-pubertal and children with autism and controls. Croonenberghs et al. (2010) reported testosterone levels over time with nine measurements in affected children versus healthy controls, whereby all measurements showed, in contrast to the general consensus, higher testosterone levels in healthy controls than those in affected children.

Notably, these analyses attempted to capture evidence concerning the two main products of a potentially increased 17, 20-lyase activity with higher levels of DHEA and androstenedione. The latter is later transformed via 17betaHSD to testosterone (Figure 1). Here, we summarize evidence from eight studies encompassing over 1000 measurements from 321 boys with autism (Table 1). However, it must be kept in mind that there could be a potential publication bias, which might be due to studies, such as those discussing extreme male brain theory, implying that levels of androgens are high in autism [40]. Corresponding funnel plots lessen this concern by showing a relatively symmetric distribution across the board, even though the scattering is strong, especially in girls. However, in girls, evidence is much sparser, with only three studies identified, with a total sample size of 64 girls with autism (Table 1). Except for testosterone levels in boys, the random effect size model (a random effect size model was used in the presence of highly heterogeneous results as seen by a high I^2^ and Q) shows significant effect sizes for all measured hormones. The effect size itself is relatively constant and high in both genders with an SMD (standardized mean difference) of 2.18 and 2.10 for androstenedione/diol in boys and girls, respectively, and an SMD of 1.42 and 1.46, respectively, for DHEA(-S/C). Therefore, different aspects, such as severity of impairment or development status, must be considered (see Supplementary Table S1). In summary, higher levels of DHEA, androstenedione/androstenediol, and testosterone are implied and, as such, an increased 17, 20-lyase activity seems to prevail. Causes of the detected pattern might be increased oxidative stress, resulting in an increased 17, 20-lyase activity that catalyzes the activity of adrenal P450c17 through p38α [7,49,50,51,52]. Involvement of the tryptophan metabolism, which has an effect via mitochondria on melatonin as a reactive oxygen species inhibitor, might be a further explanation for increased oxidative stress and, as a consequence, increased 17, 20-lyase activity [39]. When further addressing the cause of altered androgens, it was shown in parallel measurements of a unique sample that not only were DHEA and testosterone increased, but, in addition, in plasma measurements, glutathione, plasma cysteine, plasma methionine, serum cystathionine, and serum homocysteine were all significantly decreased [44]. However, there is still much uncertainty regarding the potential mechanism involved. It has to be considered that androgens or testosterone itself do not seem to necessarily induce autism, because in congenital adrenal hyperplasia (CAH) with an excess of androgens, induction of autism does not forcefully occur. Nevertheless, steroids exert organizational and activating actions during brain development [53,54] and modulate neurotransmission either by directly interacting with neurotransmitter receptors or by genomic mechanisms [27,48,55,56]. Thus, examination of steroid hormones, and androgens in particular, seems justified [8]. Recent developments using biochemical tools, such as transcriptomics, proteomics, and cellular models, will pave the way to gaining new insights into the underlying pathological pathways [29].

## 4. Conclusions

There are clear indicators that androgens are affected in boys and girls with autism. Here, we present relatively straightforward evidence that DHEA, androstenedione, and testosterone levels are higher in such children and, as a consequence, an increased activity of 17, 20-lyase is present. As this enzyme is upregulated by oxidative stress, therapeutic targeting might result from inhibiting 17, 20-lyase activity via a reduction in oxidative stress. Oxidative stress has previously been noted numerous times to be involved in the pathogenesis of autism and might yield high androgen levels [50,57]. It was shown that unexplained hyperandrogenic oligoanovulation as a main feature of polycystic ovary syndrome (PCOS) and P450c17 phosphorylation selectively increases 17, 20-lyase activity and androgen biosynthesis [50]. Furthermore, mitogen-activated protein kinase 14 (MAPK14, p38α) as the kinase responsible for enhancing 17, 20-lyase activity through P450c17 phosphorylation was increased under oxidative stress, yielding increased 17, 20-lyase activity through oxidant-sensitive p38α signaling pathways. In addition, it significantly induced dehydroepiandrosterone production and increased p38α phosphorylation and activation [50]. This might explain hyperandrogenism reducing oxidative stress, resulting in a reduction in social impairment as a consequence. As previously suggested, metformin, which is already efficiently being used in women with hyperandrogenism due to PCOS, reduces oxidative stress and hyperandrogenism and, as such, this might be one pharmacological target [58].

## 5. Ethics and Dissemination Policy

### 5.1. Developmental STAGE

As development is an important determinant of steroid hormones, age is important in studies examining androgens in ASD cohorts. Even more appropriate are Tanner stages as reference for pubertal status, whereby, for example, the studies conducted by Croonenberghs et al. (2010), El-Baz et al. (2014), and Tordjman (1995) used these to match with controls. Janšáková only analyzed prepubertal boys, while Gasser et al. analyzed postpubertal boys and matches for age and BMI. Geier used specific reference values.

### 5.2. Severity of Impairment

For example, Gasser et al. (2018) clearly distinguished between boys with early infantile autism and Asperger syndrome according to DSM-IV. A different pattern of dysregulation of androgens in Asperger versus early infantile autism was identified. The following study by Janšáková et al. also relied on the DSM-V diagnostic criteria. In summary, severity of impairment plays a role, and associations between increased androgens and severity were made, e.g., by Takagishi et al. 2010. Effect sizes calculated here are presented for prepubertal and postpubertal groups where possible, but they do not control for severity of impairment.

### 5.3. Gender

Autism is more prevalent in boys than in girls. As a consequence, evidence from girls is much sparser than that from boys. Only three studies examining girls could be analyzed; the study conducted by Geier and Geier (2006) with only two girls was not included.

### 5.4. Comorbidities and Medication Use

Comorbidities can play a key role, for example, in fragile X syndrome, where increased androgen levels show a clear association, and fragile X syndrome is associated with autism. As a consequence, there are interaction effects.

### 5.5. Diagnostic Differences throughout History and between Samples

Changing diagnostic criteria for ASD likely impacts the conclusions that can be drawn about steroid hormones in autism. For example, the studies conducted by Gasser et al. distinguished early infantile autism and Asperger syndrome according to DSM -IV (American Psychiatric Association, 1994), while later studies have included children diagnosed with autism spectrum disorders. Thus, earlier studies might have included a narrower range of children and adolescents with autism.

### 5.6. Sample Size

It should also be noted that most sub-samples analyzed have relatively small sample sizes of around twenty (Table 1), and the small sub-samples limit the generalizability of each individual study; as such, very high effect sizes, e.g., detection in the sub-sample of girls with autism by Gasser et al. 2020, should be critically questioned.

### 5.7. Methodical Problems

Here, analyses from blood, urine, and saliva fluid are presented. However, with all forms of measurements, errors can occur. For example, when performing GC–MS from urine, a bias can result when first measuring samples from children with autism and then those from a cohort. During the measurement process, sensitivity loss can result in a systematic measurement error. Thus, measurements from children with autism and controls should be analyzed separately. However, studies normally do not report such measurement procedures.

## Figures and Tables

**Table 1:** Overview of the analyzed studies.

Study/Author	Number of Patients and Description of Inclusion Criteria								Methods and Measured Outcome	Major Findings	Notes
	Boys				Girls						
	Autism		Control		Autism		Control				
	Prepubertal	Postpubertal	Prepubertal	Postpubertal	Prepubertal	Postpubertal	Prepubertal	Postpubertal			
TORDJMAN ET AL. 1995	31N/A	10N/A	8 healthy children	11 healthy children					Blood (plasma), Testosterone and DHEA-S	No alterations of Testosterone and DHEA-S	no significant increase neither in the prepubertal nor in the postpubertal group of children with autism as compared to ten healthy controls
		14 Sixteen consecutive pre-pubertal age children (</=11 years old; mean +/- SD: 5.9 +/- 2.1 years old)	Age- and sex- specific reference values from LabCorp		2 Sixteen consecutive pre-pubertal age children (</=11 years old; mean +/- SD: 5.9 +/- 2.1 years old)		Age- and sex- specific reference values from LabCorp		Blood samples	Significantly increased levels of serum/plasma DHEA and serum total Testosterone relative to the age- and sex-specific normal laboratory reference ranges were observed.	There was no control group—results were reported as percent of mean reference value
		59 children 10.8 ± 4.1 (34 with Autism and 36 with Asperger syndrome and PDD-NOS according to DSM-IV criteria, >/= 6 years-old)	Age- and sex- specific reference values from LabCorp		11 children 10.8 ± 4.1 (34 with Autism and 36 with Asperger syndrome and PDD-NOS according to DSM-IV criteria, >/= 6 years-old)		Age- and sex- specific reference values from LabCorp		Blood samples, serum Testosterone, serum free Testosterone, % free Testosterone, DHEA, Androstendione, morning blood samples collected afer overnight fast	Affected subjects showed significantly increased relative mean levels for: serum Testosterone (158%), serum free Testosterone (214%), percent free Testosterone (121%), DHEA (192%), and Androstenedione (173%). Additionally, at least one of the androgen attributes examined exceeded its recognized laboratory age- and sex-specific reference range in 81.4% (57 of 70) of the patients examined. With respect to their age- and sex-specific reference ranges, females had significantly higher overall mean relative Testosterone and relative free Testosterone levels than males.	There was no control group—results were reported as percent of mean reference value
CROONENBERGHS ET AL. 2010		18 DSM-IV criteria to make the diagnosis of autism.		22 healthy volunteers					Blood samples, the serum Testosterone concentration on 9 consecutives time points between 08.00 AM and 12.00 AM	The total Testosterone concentration was significantly lower in the autism group compared to the group of healthy controls.	
MAJEWSKA ET AL. 2013	23 age group 3–4 years DSM-IV	19 age group 7–9 years	20 age group 3–4 years DSM-IV	17 age group 7–9 years	22 age group 3–4 years DSM-IV	13 age group 7–9 years	16 age group 3–4 years DSM-IV	18 age group 7–9 years	salivary levels of 22 steroids	Children with autism had significantly higher Androstenediol, DHEA, Androsterone and their polar conjugates), indicative of precocious adrenarche and predictive of early puberty	
EL-BAZ ET AL. 2014	30 (DSM-IV), 12 (40%) had mild to moderate autism and 18 (60%) had severe autism.		20 sex- and pubertal-stage-matched children and adolescents						Blood (serum), serum free Testosterone, DHEA, Δ4-Androstenedione (Δ4-A).	11 showed higher free Testosterone levels, 9 had high DHEA, 12 had high Δ4-A and 8 children showed an elevation of all androgen levels, an association was detected between disease severity and androgen levels.	
GASSER ET AL. 2019		41 20 boys with Asperger syndrome, 21 boys with Kanner’s syndrome		41 matched for age, weight, and height					comprehensive steroid hormone metabolite analysis via gas chromatography–mass spectrometry from urine probes controlled for creatinine excretion	Higher levels of most steroid metabolites were detected in boys with Kanner’s syndrome and Asperger syndrome compared to their matched controls. These differences were more pronounced in affected individuals with Kanner’s syndrome versus Asperger syndrome.	A specific and unique pattern of alteration of Androsterone, Etiocholanolone, Progesterone, Tetrahydrocortisone, and Tetrahydrocortisol was identified in boys with Kanner’s syndrome and Asperger syndrome.
GASSER ET AL. 2020						16 Sixteen autistic girls (BMI 17.4 ± 2.8; average age 14.3 + 4.2 years)		16 matched control cohort for age, weight and height (BMI 16.8 ± 2.4; average age 14.4 ± 4 years)	Urine—MS-GC		
JANSAKOVA ET AL. 2020	86 DSM-V		24 age and sex-matched neurotypical control group						Blood—MS-GC		
TOTAL PER CATEGORY	170	161	94	69	35	29	16	34			
TOTAL PER CLASS	331		163		64		50				

## Data Availability

Data is applicable on request.

## Supplementary Materials

The following are available online at https://www.mdpi.com/article/10.3390/ijms222212324/s1, Table S1: Issues and considerations in performed androgen studies in children with autism.
