# Supplementary material for: Hyperandrogenism? Increased 17, 20-Lyase Activity? A Metanalysis and Systematic Review of Altered Androgens in Boys and Girls with Autism
Source: Int J Mol Sci. 2021 Nov 15;22(22):12324. doi: 10.3390/ijms222212324 (PMC8620117; doi:10.3390/ijms222212324)
Supplement: Supplementary file 1 [file ijms-22-12324-s001.zip › ijms-1458037-supplementary.pdf]

STUDY/AUTHOR	NUMBER OF PATIENTS AND DESCRIPTION OF INCLUSION CRITERIA								METHODS AND MEASURED OUTCOME	MAJOR FINDINGS	NOTES
	Boys				Girls						
	Autism		Control		Autism		Control				
	Prepubertal	Postpubertal	Prepubertal	Postpubertal	Prepubertal	Postpubertal	Prepubertal	Postpubertal			
**TORDJMAN ET AL. 1995**	31<br>N/A	10<br>N/A	8<br>healthy children	11<br>healthy children					Blood (plasma), Testosterone and DHEA-S	No alterations of Testosterone and DHEA-S	no significant increase neither in the prepubertal nor in the postpubertal group of children with autism as compared to ten healthy controls
**GEIER & GEIER, 2006**		14<br>Sixteen consecutive pre-pubertal age children (</=11 years old; mean +/- SD: 5.9 +/- 2.1 years old)	Age- and sex- specific reference values from LabCorp		2<br>Sixteen consecutive pre-pubertal age children (</=11 years old; mean +/- SD: 5.9 +/- 2.1 years old)		Age- and sex- specific reference values from LabCorp		Blood samples	Significantly increased levels of serum/plasma DHEA and serum total Testosterone relative to the age- and sex-specific normal laboratory reference ranges were observed.	There was no control group – results were reported as percent of mean reference value
**GEIER & GEIER, 2007**		59<br>children 10.8 ± 4.1 (34 with Autism and 36 with Asperger syndrome and PDD-NOS according to DSM-IV criteria, >/= 6 years-old)	Age- and sex- specific reference values from LabCorp		11<br>children 10.8 ± 4.1 (34 with Autism and 36 with Asperger syndrome and PDD-NOS according to DSM-IV criteria, >/= 6 years-old)		Age- and sex- specific reference values from LabCorp		Blood samples, serum Testosterone, serum free Testosterone, % free Testosterone, DHEA, Androstendione, morning blood samples collected afer overnight fast	Affected subjects showed significantly increased relative mean levels for: serum Testosterone (158%), serum free Testosterone (214%), percent free Testosterone (121%), DHEA (192%), and Androstenedione (173%). Additionally, at least one of the androgen attributes examined exceeded its recognized laboratory age- and sex-specific reference range in 81.4% (57 of 70) of the patients examined. With respect to their age- and sex-specific reference ranges, females had	There was no control group – results were reported as percent of mean reference value

										significantly higher overall mean relative Testosterone and relative free Testosterone levels than males.	
**CROONENBERGHS ET AL. 2010**		18<br><br>DSM-IV criteria to make the diagnosis of autism.		22<br><br>healthy volunteers					Blood samples, the serum Testosterone concentration on 9 consecutives time points between 08.00 AM and 12.00 AM	The total Testosterone concentration was significantly lower in the autism group compared to the group of healthy controls.	
**MAJEWSKA ET AL. 2013**	23<br><br>age group 3-4 years DSM-IV	19<br><br>age group 7-9 years	20<br><br>age group 3-4 years DSM-IV	17<br><br>age group 7-9 years	22<br><br>age group 3-4 years DSM-IV	13<br><br>age group 7-9 years	16<br><br>age group 3-4 years DSM-IV	18<br><br>age group 7-9 years	salivary levels of 22 steroids	Children with autism had significantly higher Androstenediol, DHEA, Androsterone and their polar conjugates), indicative of precocious adrenarche and predictive of early puberty	
**EL-BAZ ET AL. 2014**	30<br><br>(DSM-IV), 12 (40%) had mild to moderate autism and 18 (60%) had severe autism.		20<br><br>sex- and pubertal-stage-matched children and adolescents						Blood (serum), serum free Testosterone, DHEA, Δ4-Androstenedione (Δ4-A).	11 showed higher free Testosterone levels, 9 had high DHEA, 12 had high Δ4-A and 8 children showed an elevation of all androgen levels, an association was detected between disease severity and androgen levels.	
**GASSER ET AL. 2019**		41<br><br>20 boys with Asperger syndrome, 21 boys with Kanner's syndrome		41<br><br>matched for age, weight, and height					comprehensive steroid hormone metabolite analysis via gas chromatography–mass spectrometry from urine probes controlled for creatinine excretion	Higher levels of most steroid metabolites were detected in boys with Kanner's syndrome and Asperger syndrome compared to their matched controls. These differences were more pronounced in affected individuals with Kanner's syndrome versus Asperger syndrome.	A specific and unique pattern of alteration of Androsterone, Etiocholanolone, Progesterone, Tetrahydrocortisone, and Tetrahydrocortisol was identified in boys with Kanner's syndrome and Asperger syndrome.

**GASSER ET AL. 2020**						16<br><br>Sixteen autistic girls (BMI 17.4 ± 2.8; average age 14.3 + 4.2 years)		16<br><br>matched control cohort for age, weight and height (BMI 16.8 ± 2.4; average age 14.4 ± 4 years)	Urine – MS-GC
**JANSAKOVA ET AL. 2020**	86<br><br>DSM-V		24<br><br>age and sex-matched neurotypical control group						Blood – MS-GC
**TOTAL PER CATEGORY**	170	161	94	69	35	29	16	34	
**TOTAL PER CLASS**	331		163		64		50		
